# Aberrant CD25 and Increased CD123 Expression Are Common in Acute Myeloid Leukemia with *KMT2A* Partial Tandem Duplication and Are Associated with *FLT3* Internal Tandem Duplication

**DOI:** 10.3390/cancers18020282

**Published:** 2026-01-16

**Authors:** Qing Wei, Guilin Tang, Shaoying Li, Sa A. Wang, Pei Lin, Wei Wang, Sanam Loghavi, Wei J. Wang, L. Jeffrey Medeiros, Jie Xu

**Affiliations:** Department of Hematopathology, MD Anderson Cancer Center, The University of Texas, Houston, TX 77030, USA; qwei1@mdanderson.org (Q.W.); gtang@mdanderson.org (G.T.); sli6@mdanderson.org (S.L.); swang5@mdanderson.org (S.A.W.); peilin@mdanderson.org (P.L.); wwang13@mdanderson.org (W.W.); sloghavi@mdanderson.org (S.L.); wwang20@mdanderson.org (W.J.W.); ljmedeiros@mdanderson.org (L.J.M.)

**Keywords:** AML, *KMT2A* PTD, *FLT3* ITD, immunophenotype, CD25, CD123

## Abstract

Background: *KMT2A* partial tandem duplication (PTD) occurs in approximately 5–10% of acute myeloid leukemia (AML) cases and is associated with poor prognosis. While its cytogenetic and molecular features are well described, the immunophenotypic characteristics of AML with *KMT2A*-PTD remain incompletely defined. Methods: We identified 47 cases of AML with *KMT2A*-PTD by optical genome mapping. All cases underwent flow cytometric immunophenotypic analysis and next-generation sequencing using an 81-gene panel. Results: The cohort included 32 men and 15 women with a median age of 67 years (range, 19–87). Thirty-eight cases were de novo AML, and nine were secondary to myelodysplastic syndrome and/or myeloproliferative neoplasm. Most cases (93%) demonstrated a normal or non-complex karyotype. The most frequent mutations involved *FLT3-ITD* (47%), *DNMT3A* (43%), and *RUNX1* (23%). Thirty-one cases (66%) were granulocytic, while 16 (34%) showed granulocytic and/or monocytic differentiation. Blasts uniformly expressed HLA-DR and frequently expressed CD117 (91%) and CD34 (79%). Increased expression of CD123 (74%) and CD117 (43%) and decreased expression of HLA-DR (74%) and CD38 (69%) were common. Aberrant CD25 expression was observed in 51% of cases. Increased CD123 and aberrant CD25 expression were significantly associated with *FLT3*-ITD mutations (both *p* < 0.0001) but not with other recurrent mutations. There was no correlation between *FLT3*-ITD mutation and expression levels of CD117, CD38 or HLA-DR (all *p* > 0.05). Conclusions: AML with *KMT2A*-PTD shows distinctive immunophenotypic features with increased CD123 and aberrant CD25 expression, both associated with *FLT3*-ITD. These markers may have diagnostic and therapeutic relevance in this AML subtype.

## Simple Summary

Acute myeloid leukemia (AML) with *KMT2A* partial tandem duplication (PTD) is associated with poor prognosis, but its immunophenotype has not been well defined. We studied 47 AML cases with *KMT2A*-PTD confirmed by optical genome mapping. Most cases showed a relatively simple karyotype and frequent mutations in *FLT3*-ITD, *DNMT3A*, and *RUNX1* by next-generation sequencing. Immunophenotypic analysis revealed that blasts commonly expressed CD34, CD117 and HLA-DR, with frequently aberrant CD25 and increased CD123 expression. CD25 and increased CD123 expression were significantly associated with *FLT3*-ITD mutations, but not with other common mutations. These findings identify distinctive immunophenotypic features of AML with *KMT2A*-PTD and suggest that CD25 and CD123 may serve as useful biomarkers and potential therapeutic targets in this AML subtype.

## 1. Introduction

*KMT2A* (previously known as *MLL*) partial tandem duplication (PTD) is an intragenic duplication that typically spans exon 2 or 3 through exon 6 or exons 8–11 [1,2]. This alteration occurs in approximately 5–10% of acute myeloid leukemia (AML) cases [1,3]. AML with *KMT2A*-PTD (*KMT2A*-PTD AML) is frequently associated with a normal karyotype or trisomy 11 [4,5] and these neoplasms often carry *FLT3* internal tandem duplication (ITD) and mutations of *DNMT3A* and *RUNX1* [6,7,8]. Patients with *KMT2A*-PTD AML have a poor prognosis [9] and therefore identifying new therapeutic targets for these patients is critical to improve outcomes. *KMT2A*-PTD drives leukemogenesis through epigenetic activation of HOX genes [6,10,11]. *KMT2A*-PTD mice showed increased histone H3/H4 acetylation and H3 lysine 4 (Lys4) methylation within the promoter regions of HOX genes, resulting in sustained transcriptional activation and overexpression [10]. Therefore, the HOX gene expression in *KMT2A*-PTD AML may have potential therapeutic implications for menin inhibitors [12].

Understanding the immunophenotype of *KMT2A*-PTD AML cases may help to identify potential therapeutic targets. Although the cytogenetic and molecular landscape of *KMT2A*-PTD AML has been well described, its immunophenotypic features remain poorly characterized. An early study reported that ~27% of *KMT2A*-PTD AML cases show monocytic differentiation (M4 or M5 by FAB classification) [1,7]. Gonen and colleagues suggested an association between *KMT2A*-PTD and aberrant CD25 expression in AML [13], but this study was small and did not have a correlative study with genetic data. CD25 (IL-2Rα) is a component of the interleukin-2 (IL-2) receptor complex that mediates T-cell proliferation, differentiation and immune regulation [14]. Immunophenotype-genotype associations may be helpful for early recognition of genetic alterations such as *KMT2A* rearrangement [15] and possibly *KMT2A*-PTD. Generally, AML blasts are most often CD34+ CD117+ HLA-DR+, but *KMT2A*-rearranged AML cases are characterized by CD34- CD117+ HLA-DR+ blasts and monocytic differentiation [15]. It is unknown if AML cases with *KMT2A*-PTD have an immunophenotype similar to *KMT2A*-rearranged cases.

*KMT2A*-PTD is cryptic to conventional cytogenetic methods including karyotypic and fluorescence in situ hybridization (FISH) analysis, making detection challenging. Newer technologies, however, such as DNA or RNA next-generation sequencing (NGS) and optical genome mapping (OGM), can detect *KMT2A*-PTD [3,16,17,18]. OGM is a novel genome-wide structural variant detection platform that provides high-resolution analysis of the human genome, enabling identification of balanced and unbalanced rearrangements as well as intragenic gene duplications. In hematologic malignancies, studies have demonstrated a high degree of concordance between OGM and standard cytogenetic analysis, with OGM detecting 95–100% of cytogenetic abnormalities identified by standard cytogenetic analysis. Importantly, OGM also provides additional clinically relevant cytogenomic information in approximately 15–30% of patients beyond that obtained by conventional cytogenetic methods [3,16,19,20,21,22,23]. We recently reported the utility of OGM for detecting cryptic *KMT2A*-PTD in hematologic malignancies [3], during which we identified a number of cases of AML with *KMT2A*-PTD. In the present study, we performed a comprehensive immunophenotypic analysis of this cohort of *KMT2A*-PTD AML cases. We focus on potential biomarkers and their association with genetic abnormalities in cases of AML with *KMT2A*-PTD.

## 2. Materials and Methods

### 2.1. Case Selection and Data Collection

The study cohort included 47 patients with AML harboring *KMT2A*-PTD identified by OGM at our institution between 1 November 2022 and 30 April 2025. The diagnosis of AML was based on morphologic, cytochemical, immunophenotypic, molecular and cytogenetic findings according to the World Health Organization Classification (WHO; 5th edition) and the International Consensus Classification (ICC; 2022) [24,25]. The detection of *KMT2A*-PTD by OGM has been previously described [3]. The clinicopathological data for a subset of patients in the current study have been reported previously, but without immunophenotypic characterization. Briefly, OGM was performed on fresh peripheral blood (PB) or bone marrow (BM) aspirate specimens following the manufacturer’s protocol (Bionano Genomics, San Diego, CA, USA) [18]. Ultra-high-molecular-weight genomic DNA was extracted, labeled and imaged using the Bionano Saphyr system (San Diego, CA, USA). Data analyses were conducted with the Rare Variant Analysis Pipeline in Bionano Access (version 1.7.2), focusing on clinically relevant genes and loci. *KMT2A*-PTD was identified as an intragenic insertion and/or duplication within *KMT2A*. Clinical data were obtained through a comprehensive review of electronic medical records. The study was approved by the Institutional Review Board.

### 2.2. Morphological Assessment

Routine hematoxylin and eosin (H&E) histologic sections of BM trephine biopsy and clot specimens and Wright–Giemsa-stained aspirate smears were reviewed. A 500 nucleated cell differential count was performed. A myeloperoxidase (MPO) cytochemical stain was performed in all cases and non-specific esterase (NSE) stain was performed selectively in cases with monocytic differentiation as assessed by morphological evaluation.

### 2.3. Flow Cytometric Immunophenotypic Analysis

Flow cytometric immunophenotyping was performed on fresh PB or BM aspirate specimens using a standard stain/lyse/wash technique (PharmLyse, BD Biosciences, Franklin Lakes, NJ, USA) as part of routine clinical workup, as previously described [19]. The antibody panel included: CD2, CD3 (cytoplasmic and surface), CD4, CD5, CD7, CD13, CD14, CD15, CD19, CD25, CD33, CD34, CD36, CD38, CD45, CD54, CD56, CD64, CD117, CD123, CD133, HLA-DR, MPO and TDT (The detailed product information is listed in Supplemental Table S1). For each analysis, a minimum of 200,000 events were acquired on FACSCanto II instruments (BD Biosciences), in which standardization was maintained using CS&T beads (BD Biosciences), with an emphasis on comparable cross-platform performance. Data was analyzed using FCS Express flow cytometry software version 6 (De Novo Software, Pasadena, CA, USA). Blasts were identified using a CD45 dim gate with low side scatter, confirmed by back-gating with CD34 and CD117. The markers are expressed on a bi-exponential scale (a data-transformation and display scale that combines linear behavior near zero with log-like behavior at larger magnitudes, on both the positive and negative sides of zero). The expression of each marker of the population of interest is measured by median fluorescence intensity (MFI; the actual read by FCS Express flow cytometry software). For most markers, positivity was defined as expression in ≥20% of target cells (20–80% for partial positivity and ≥80% for uniform positivity), using background fluorescence (internal negative population or unstained control) as reference. Antigen expression intensity (increased or decreased) was assessed relative to normal/reactive cells. Increased CD123 expression was defined as an MFI ≥ 1100 [26]. CD25 is typically negative on normal myeloblasts; in this study, CD25 expression was considered positive at a threshold of ≥10% of blasts, based on literature [20,21]. Cases with CD25 expression on 10–80% of myeloblasts were considered partially positive, where those with CD25 expression in more than 80% blasts were considered as uniformly positive.

### 2.4. Chromosomal Banding Assay

Conventional chromosomal banding analysis was performed as part of the diagnostic workup. Twenty Giemsa-banded metaphase cells were analyzed, and results were reported according to the 2020 International System for Human Cytogenomic Nomenclature (ISCN). A complex karyotype was defined as ≥3 unrelated chromosomal abnormalities in the absence of other class-defining recurrent genetic alterations and excluding hyperdiploid karyotypes with ≥3 trisomies (or polysomies) without structural abnormalities.

### 2.5. DNA Next-Generation Sequencing and FLT3-ITD and FLT3-TKD Analysis

Next-generation sequencing (NGS) analysis was performed as previously described [22]. NGS was performed using a laboratory-developed panel encompassing 81 genes known to be recurrently altered in myeloid neoplasms (Supplementary Table S2). The assay was designed to capture either complete coding regions or selected mutational hotspots of these genes. Genomic DNA (500 ng) was used for library construction with hybridization-based enrichment of regions of interest, followed by paired-end sequencing on an Illumina NextSeq instrument (Illumina, San Diego, CA, USA). Sequence reads were initially processed for base calling with Illumina bcl2fastq software (v2.20.0), and alignment to the human reference genome (GRCh37/hg19) was performed using Sequence Analysis Viewer version 2.4.7. Downstream data processing, including generation of BAM and VCF files, was conducted using an internally developed analysis and reporting pipeline (Oncoseek v2.2.0.1) to facilitate variant interpretation and annotation. For clinical reporting, this assay achieves a limit of detection of approximately 1% variant allele frequency for single nucleotide variants and 2–3% for small insertions and deletions. Only variants supported by a sequencing depth of at least 250 reads were considered reportable, with most target regions routinely exceeding 1000× coverage. The assay is optimized for detection of point mutations and short indels up to 52 base pairs in length, while larger structural variants and copy number alterations are not reliably captured by this method.

*FLT3*-ITD and *FLT3*-tyrosine kinase domain (TKD) mutations were assessed by multiplex fluorescent polymerase chain reaction (PCR) followed by capillary electrophoresis on DNA extracted from PB or BM aspirate samples, as previously described [23].

### 2.6. Statistics

Statistical analyses were performed using GraphPad Prism 9 software (GraphPad Software, Boston, MA, USA). Categorical variables were analyzed using the χ^2^ test. The Mann–Whitney U test was used to compare differences in MFIs of CD123, CD38, CD117 and HLA-DR, between cases with and without *FLT3*-ITD, *DNMT3A*, *RUNX1* or *FLT3*-TKD mutations. Overall survival (OS) was defined as the interval from the date of initial diagnosis to the date of death or last follow-up. Survival analyses were performed using the Kaplan-Meier method and differences between groups were assessed using the log-rank test. A *p* value of less than 0.05 was considered statistically significant.

## 3. Results

### 3.1. Clinical and Pathological Features

**Figure 1:**
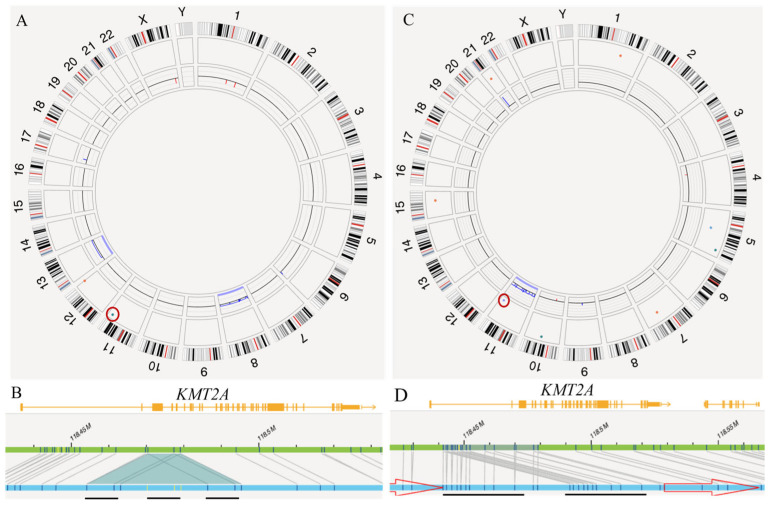
Cryptic *KMT2A* partial tandem duplication (PTD) identified by optical genome mapping (OGM). (**A**). Circos plot shows whole-chromosome gains of chromosomes 8 and 13 and a focal structural abnormality at 11q23 (red circle). (**B**). Genome Browser visualization demonstrates an intragenic insertion and duplication within *KMT2A*, starting near exon 2, with a VAF of 0.98. The OGM data are represented with specific visual elements to aid interpretation. The orange track represents the *KMT2A* gene structure, with vertical bars indicating exons and horizontal lines indicating introns. The blue track depicts the alignment of the sample OGM consensus map to the reference genome (green track). Manual examination reveals an “insertion” and duplication of the same fragment, resulting in a triplication. The triplicated sequence is underlined by three short black lines at the very bottom. (**C**). Circos plot shows a whole-chromosome gain of chromosome 11 and a similar focal abnormality at 11q23 (red circle). (**D**). Genome Browser visualization demonstrates an intragenic duplication within *KMT2A*, also starting near exon 2, with a VAF of 0.95. The duplicated sequence, verified by manual examination, is underlined by two short black lines at the very bottom.

The cohort included 32 men and 15 women with a median age of 67 years (range, 19–87 years) at diagnosis (Table 1). Thirty-eight cases arose de novo (primary) AML and nine cases occurred in patients with a history of myelodysplastic syndrome, chronic myelomonocytic leukemia or myeloproliferative neoplasm (secondary). Of the 45 cases with available karyotype results, 42 (93%) showed a normal or non-complex karyotype. *KMT2A*-PTD was cryptic by conventional chromosomal analysis. OGM identified *KMT2A* PTD as either an intragenic insertion (n = 43) or an intragenic duplication (n = 4) involving chromosome 11q23. Examples of cryptic *KMT2A*-PTD detected as an insertion and as a duplication are illustrated in Figure 1A,B and Figure 1C,D, respectively. The most common mutations were *FLT3*-ITD (47%, n = 22), *DNMT3A* (43%, n = 20) and *RUNX1* (23%, n = 11). *FLT3*-TKD mutations were identified in 7 (15%) cases (Table 1). The mutation profile of all cases is shown in Supplemental Table S3.

**Figure 2:**
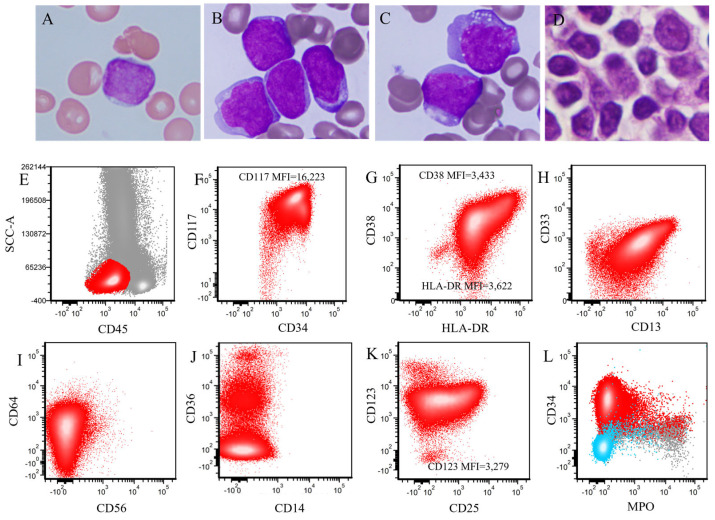
Morphological and immunophenotypic findings of a representative granulocytic *KMT2A*-PTD AML case (without monocytic differentiation) (**A**–**C**). Peripheral blood (**A**) and bone marrow aspirate smears (**B**,**C**) show large blasts, with round to slightly irregular nuclei, fine chromatin, and small amounts of cytoplasm. (**D**). The bone marrow core biopsy shows sheets of blasts. (**A**–**D**), original magnification ×1000. (**E**–**L**). Flow cytometric immunophenotyping shows increased blasts (red dots). The blasts are positive for CD34, CD117 (increased), HLA-DR (decreased), CD38 (decreased), CD13 (partial), CD33 (partial), CD64 (partial/dim), CD36 (small subset), CD25 (partial), CD123 (increased), and are negative for CD56, CD14 and MPO. Light blue dots indicate the background lymphocytes (**L**).

**Figure 3:**
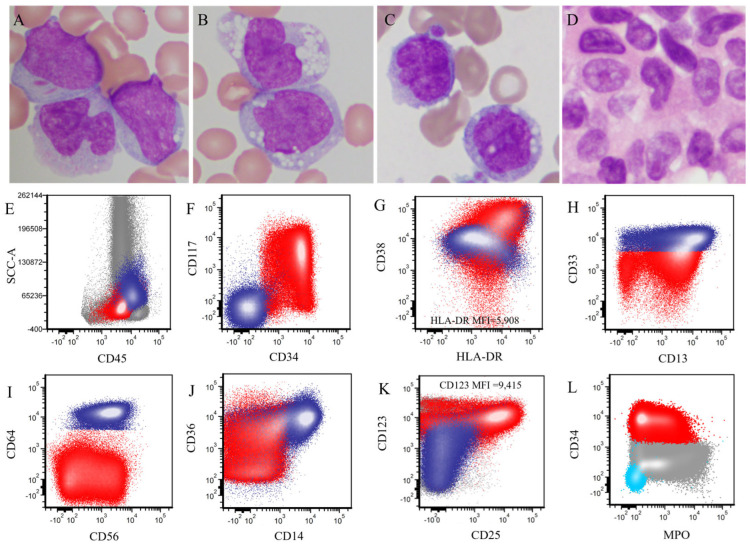
Morphological and immunophenotypic findings of a representative *KMT2A*-PTD AML case with monocytic differentiation. (**A**–**C**). Peripheral blood (**A**,**B**) and bone marrow aspirate smear (**C**) show large blasts with irregular nuclei, fine chromatin, and small to moderate amounts of cytoplasm. Auer rods are identified. Some blasts show folded nuclear contours and vacuolated cytoplasm (**B**,**C**), consistent with monocytic differentiation. (**D**). The bone marrow core biopsy shows blasts with round or folded nuclear contours. (**A**–**D**), original magnification ×1000. (**E**–**L**). Flow cytometric immunophenotyping shows increased blasts (red dots) and monocytic cells (dark blue dots). The myeloblasts are positive for CD34, CD117, CD38, HLA-DR (decreased), CD13, CD33, CD56 (partial), CD36 (partial), CD25, CD123 (increased) and MPO, and are negative for CD64 and CD14. The monocytic cells (dark blue dots) are positive for CD38, HLA-DR (partial/decreased), CD33 (bright), CD13 (partial), CD64 (bright), CD56 (partial), CD36, CD14 and CD123 (partial), and are negative for CD34, CD117 and CD25. Light blue dots indicate the background lymphocytes (**L**).

In BM aspirate smears, the median percentage of blasts in the BM was 53% (range, 2–95%) and the median percentage of monocytes was 4% (range, 0–41%). The patient with 2% blasts was treated at outside institution before being transferred to our hospital. This patient’s bone marrow biopsy at our hospital showed residual AML (2% blasts). These *KMT2A*-PTD AML cases were categorized into two groups: 31 (66%) were granulocytic (without monocytic differentiation) and 16 (34%) exhibited granulocytic and/or monocytic differentiation, including myelomonocytic (n = 11, 23%) and monoblastic (n = 5, 11%) (Table 1). A representative granulocytic case is shown in Figure 2. The blasts were similar in PB (Figure 2A) and BM aspirate smears (Figure 2B,C): the blasts were large with round or slight irregular nuclear contours, fine chromatin, inconspicuous nucleoli, and a small amount of cytoplasm. The BM core biopsy specimens showed sheets of blasts (Figure 2D). A representative myelomonocytic case is illustrated in Figure 3. Peripheral blood and BM aspirate smears showed myeloblasts (Figure 3A), some with folded nuclei and vacuolated cytoplasm consistent with monocytic differentiation (Figure 3B,C). The BM core biopsy specimen showed immature cells with either round or folded nuclear contours (Figure 3D).

### 3.2. Immunophenotype of KMT2A-PTD AML by Flow Cytometry

**Figure 4:**
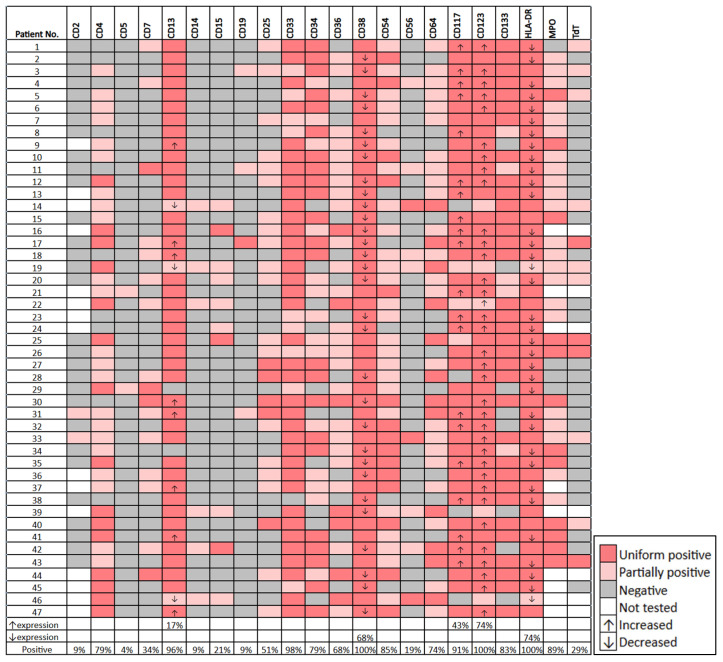
The summarized flow cytometric immunophenotype of blasts in all AML cases with *KMT2A*-PTD.

Figure 2E–L illustrates the immunophenotype of a representative granulocytic case. Figure 3E–L shows the immunophenotype of a representative myelomonocytic case. The detailed immunophenotype of blasts of all *KMT2A*-PTD AML cases (with and without monocytic differentiation) is shown in Figure 4.

In all 47 cases the blasts were positive for CD38, CD123 and HLA-DR. The blasts were positive for CD33 (n = 46, 98%), CD117 (n = 46, 98%), CD13 (n = 45, 96%), CD54 (n = 45, 96%), CD34 (n = 41, 88%), CD133 (n = 41, 88%), MPO (n = 34, 89), CD4 (n = 36, 76%), CD64 (n = 33, 71%), CD36 (n = 30, 64%) and CD25 (n = 24, 51%). The blasts were less frequently positive for other markers including CD7 (34%), TdT (32%), CD15 (21%), CD56 (19%), CD14 (13%), CD2 (9%) and CD5 (4%). The blasts also showed altered expression levels of some markers based on median fluorescence intensity (MFI): CD123 and CD117 increased in 74% and 43%, respectively and HLA-DR and CD38 decreased in 74% and 69% of cases, respectively. The mean CD123 MFI were 4093 and 995 in cases with increased vs. normal CD123 expression, respectively (*p* = 0.0007). The blasts showed aberrant CD25 expression in 24 (51%) cases: 19 uniform and 5 partial. On average, 48% and 1% of blasts were positive for CD25 in CD25+ vs. CD25-negative cases, respectively (*p* < 0.0001). Twenty (43%) cases showed both aberrant CD25 and increased CD123 expression.

**Figure 5:**
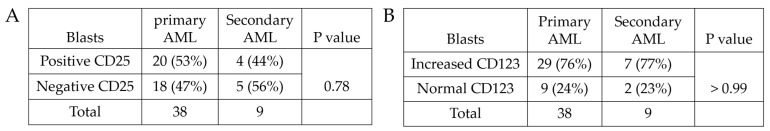
Blast expression of CD25 and CD123 in primary vs. secondary AML with *KMT2A*-PTD. (**A**,**B**). The frequency of CD25 positivity (**A**) and CD123 overexpression (**B**) did not differ between primary vs. secondary AML with *KMT2A*-PTD.

There was no significant difference in blast CD25 positivity between primary (20/38, 53%) vs. secondary (4/9, 44%) AML cases (*p* = 0.78; Figure 5A). Blast CD123 expression level was increased in 29 of 38 (76%) primary vs. 7 of 9 (77%) secondary AML cases, with no significant difference in CD123 levels between these two groups (*p* > 0.99; Figure 5B). Therefore, primary and secondary AML cases were combined for the subsequent study of CD25 and CD123.

**Figure 6:**
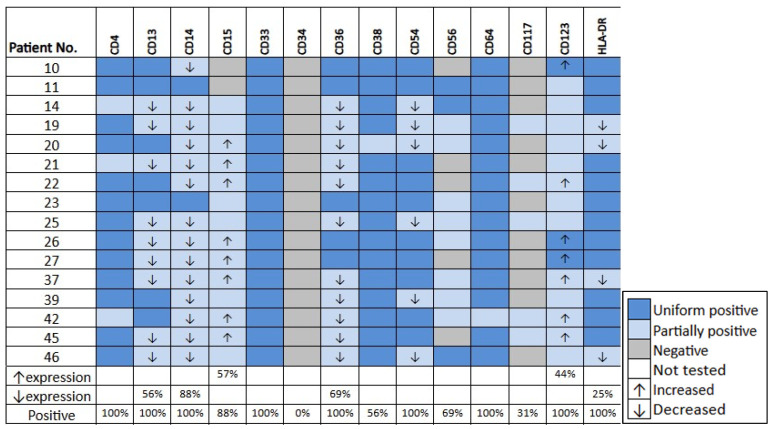
The summarized flow cytometric immunophenotype of monocytic cells in *KMT2A*-PTD AML with granulocytic and/or monocytic differentiation. The monocytic cells in all cases were negative for other markers tested (CD2, CD5, CD7, CD19, CD25 and CD133).

The detailed immunophenotype of the monocytic cells in *KMT2A*-PTD AML cases with granulocytic and/or monocytic differentiation is shown in Figure 6. The monocytic cells were negative for CD34 in all cases (n = 16) and positive for CD117 in a small subset of cases (n = 5, 31%; all partial). The monocytic cells were positive for CD4 (13 uniform and 3 partial), CD13 (9 partial and 7 uniform), CD14 (14 partial and 2 uniform), CD15 (n = 14, all partial), CD33 (16 uniform), CD36 (11 partial and 5 uniform), CD38 (15 uniform and 1 partial), CD54 (10 uniform and 6 partial), CD64 (15 uniform and 1 partial), CD123 (13 partial and 3 uniform) and HLA-DR (12 uniform and 4 partial). The monocytic cells show decreased expression levels of CD14 (n = 14, 88%), CD36 (n = 11, 69%), CD13 (n = 9, 56%), and HLA-DR (n = 4, 25%). The monocytic cells showed increased expression levels of CD15 (n = 9, 57%) and CD123 (n = 7, 44%).

### 3.3. CD25 Expression in KMT2A-PTD AML Is Associated with FLT3-ITD but Not DNMT3A, RUNX1 or FLT3-TKD Mutations

**Figure 7:**
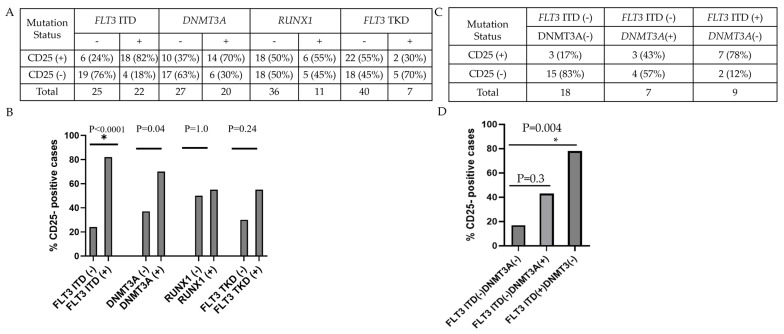
Correlative study of CD25 expression and gene mutations in *KMT2A*-PTD AML cases. (**A**,**B**). The frequency of CD25 positivity was significantly associated with *FLT3*-ITD and *DNMT3A,* but not *RUNX1* or *FLT3*-TKD mutation. (**C**,**D**). Compared with FLT3-negative *DNMT3A*-negative cases, CD25 expression was significantly increased in *FLT3*-ITD-positive *DNMT3A*-negative cases, but not in *FLT3*-ITD-negative *DNMT3A*-positive cases. * indicates statistical significance.

Blasts were more frequently positive for CD25 in *FLT3*-ITD–positive cases (18/22, 82%) than in *FLT3*-ITD–negative cases (6/25, 24%) (*p* < 0.001; Figure 7A,B). Similarly, CD25 was more often detected in *DNMT3A*-positive cases (14/20, 70%) than in *DNMT3A*-negative cases (10/27, 37%) (*p* = 0.04). In contrast, CD25 expression was not associated with *RUNX1* or *FLT3*-TKD mutation (both *p* > 0.05; Figure 7A,B). Since mutations of *FLT3*-ITD and *DNMT3A* co-existed in some *KMT2A*-PTD AML cases, i.e., 13/20 (65%) of *DNMT3A*-positive cases had *FLT3*-ITD and 13/22 (60%) of *FLT3*-ITD cases had *DNMT3A* mutations, the association between CD25 and *FLT3*-ITD or *DNMT3A* alone was also analyzed. Compared with *FLT3*-ITD-negative *DNMT3A*-negative cases, *FLT3*-ITD-positive DNMT3A-negative cases (but not *FLT3*-ITD-negative *DNMT3A*-positive cases) showed significantly increased CD25 expression (*p* = 0.004 and *p* = 0.3, respectively; Figure 7C,D).

### 3.4. Increased CD123 Expression in KMT2A-PTD AML Is Associated with FLT3-ITD but Not DNMT3A, RUNX1 or FLT3-TKD Mutations

**Figure 8:**
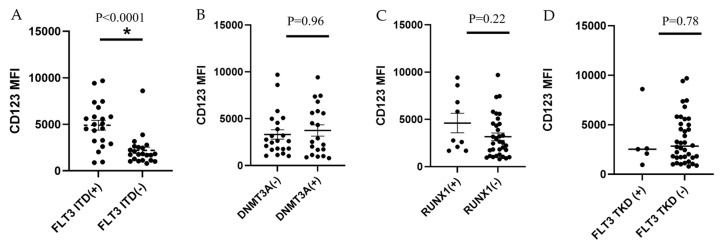
Correlative study of CD123 expression level (determined by MFI) and gene mutations in *KMT2A*-PTD AML cases. (**A**–**D**). CD123 expression level was significantly associated with *FLT3*-ITD (**A**), but not *DNMT3A* (**B**), *RUNX1* (**C**) or *FLT3*-TKD (**D**) mutations. * indicates statistical significance.

*FLT3*-ITD–positive cases showed significantly higher mean MFI of CD123, compared to *FLT3*-ITD–negative cases (mean MFI, 4901 vs. 2195; *p* < 0.0001) (Figure 8A). The mean MFI of CD123 did not significantly differ between cases with vs. without *DNMT3A*, *RUNX1*, or *FLT3-TKD* mutations (*p* = 0.96, 0.22, and 0.78, respectively; Figure 8B–D).

**Figure 9:**
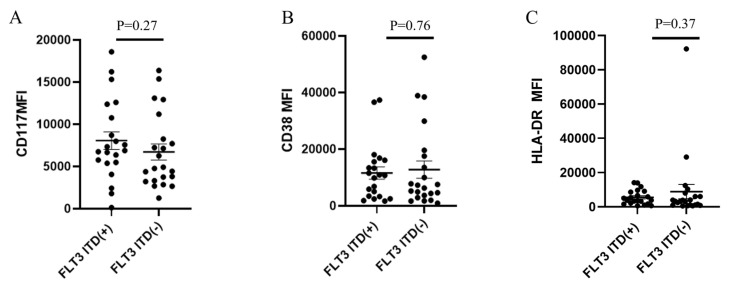
Comparison of the expression of CD117, CD38 and HLA-DR in *KMT2A*-PTD AML with vs. without *FLT3*-ITD. (**A**–**C**). *FLT3*-ITD did not affect the expression levels of CD117 (**A**), CD38 (**B**) or HLA-DR (**C**).

The mean MFIs of CD117, CD38 and HLA-DR were also compared between cases with vs. without *FLT3*-ITD, but no significant differences were found (*p* = 0.27, 0.76, and 0.37, respectively; Figure 9A–C).

### 3.5. CD25 and Increased CD123 Expression Did Not Affect the Overall Survival of Patients with KMT2A-PTD AML

To investigate the potential prognostic significance of CD25 and increased CD123 expression in *KMT2A*-PTD AML, patients were grouped based on their CD25 (positive vs. negative) and CD123 (increased vs. normal) expression status, respectively. Neither CD25 nor increased CD123 expression was significantly associated with OS (*p* = 0.09 and *p* = 0.422, respectively).

## 4. Discussion

In this study, we performed a comprehensive immunophenotypic characterization of cases of AML with *KMT2A*-PTD. We show that about one third of *KMT2A*-PTD AML cases show monocytic differentiation. The blasts in *KMT2A*-PTD AML cases are mostly CD34+ CD117+ HLA-DR+. These features differ from AML with *KMT2A*-rearrangement. The latter group has a higher frequency of monocytic differentiation (~60%), and the blasts are often CD34- CD117+ HLA-DR+ [15]. Other key immunophenotypic features of the blasts in *KMT2A*-PTD AML include increased expression levels of CD117, CD25 and CD123, decreased expression levels of CD38 and HLA-DR.

CD25 (IL-2Rα) is the alpha subunit of the interleukin-2 (IL-2) receptor that mediates T-cell proliferation, differentiation and immune regulation [14]. Normally, CD25 is expressed at high levels on regulatory T-cells (Tregs) and transiently expressed on activated lymphoid and myeloid cells. CD25 can be expressed by AML, B lymphoblastic leukemia/lymphoma (especially Ph+ or Ph-like type), adult T-cell leukemia/lymphoma, anaplastic large cell lymphoma and hairy cell leukemia [27,28,29,30]. Aberrant CD25 expression is detected in approximately 20–30% of AML cases [13,31,32] and is associated with a poorer prognosis [13,32,33]. We observed aberrant CD25 expression in about one half of AML cases with *KMT2A*-PTD. CD25 is rare in cases of AML with *KMT2A*-rearrangement [15]. Our CD25 results are consistent with an earlier report showing CD25 expression in 8 of 19 (42%) AML cases with *KMT2A*-PTD [13]. Notably, CD25 expression in our cohort was markedly enriched in *FLT3*-ITD–positive disease (82% vs. 24% in *FLT3*-ITD–negative cases), aligning with prior studies reporting an association between CD25 and *FLT3*-ITD [13,34]. Transcriptomic data from the BEAT-AML study further supports this relationship, showing a strong association between CD25 expression and *FLT3*-ITD status [35]. In the present study, among 24 cases of CD25+ *KMT2A*-PTD AML, 18 (75%) cases had *FLT3*-ITD and 14 (58%) cases had *DNMT3A* mutation. However, after removing cases carrying both *FLT3*-ITD and *DNMT3A* mutations, only *FLT3*-ITD (not *DNMT3A*) was associated with CD25 expression, indicating that the CD25 expression in *DNMT3A*-positive cases was likely due to the co-existing *FLT3*-ITD. In addition, a small subset (24%) of *FLT3*-ITD–negative *KMT2A*-PTD cases also expressed CD25, suggesting that *KMT2A*-PTD itself may contribute to CD25 upregulation independent of *FLT3*-ITD.

In addition to CD25, *KMT2A*-PTD AML cases in this cohort showed increased CD123 expression (in ~80% of cases), more often than in unselected AML cases (45–55%) [36,37]. CD123, the alpha subunit of the interleukin-3 (IL-3) receptor, plays a crucial role in hematopoietic cell survival and proliferation upon IL-3 binding [36,38]. Although CD123 is expressed at low level by most normal CD34+ myeloblasts, it is frequently overexpressed in hematological malignancies including AML, blastic plasmacytoid dendritic cell neoplasm (BPDCN), acute lymphoblastic leukemia/lymphoma, hairy cell leukemia, and etc. [38,39]. Overexpression of CD123 in AML has been associated with enhanced blast proliferation, increased cellularity and poorer patient prognosis [37]. Increased CD123 expression was associated with *FLT3-ITD* and *NPM1* mutations in AML, seen in 83% of *FLT3-ITD* and 62% of *NPM1*-mutated AML [36,40,41]. In *NPM1*-mutated AML, *FLT3*-ITD has been shown to enhance CD123 expression, leading to a synergistic effect [42]. In the current study, none of the *KMT2A*-PTD AML cases had *NPM1* mutation (Supplemental Table S3). *FLT3*-ITD-positive cases had significantly higher CD123 expression than *FLT3*-ITD-negative cases, suggesting that *FLT3*-ITD may also augment CD123 expression in the context of *KMT2A*-PTD. However, 65% of *FLT3*-ITD-negative *KMT2A*-PTD cases still showed increased CD123 expression, suggesting that *KMT2A*-PTD itself may also contribute to CD123 upregulation. A prior study using immunohistochemical staining for CD123 in AML found that a significant proportion of CD123-positive cases lacked *FLT3*-ITD and/or *NPM1* mutations [40], indicating that *FLT3*-ITD and *NPM1* mutations are not the only mechanisms underlying CD123 overexpression.

The overexpression of CD123 in hematologic malignancies provides a potential therapeutic target. The US Food and Drug Administration (FDA) and European Medicines Agency (EMA) have approved tagraxofusp, a CD123-targeted agent, for patients with BPDCN [43]. Given the overexpression of CD123 in some AMLs, CD123 has also been investigated as a therapeutic target in AML. Other agents targeting at CD123 have been developed, such as Pivekimab sunirine (IMGN632), flotetuzumab, and CD123-directed CAR T-cell therapies, some of which have shown promising clinical results [34,38]. Pivekimab sunirine, a novel CD123-targeting antibody-drug conjugate, has shown single-agent activity in relapsed/refractory AML, leading to a phase 1b/2 study of pivekimab sunirine plus azacitidine and venetoclax in patients with CD123+ AML [44]. In the presence of cytokines such as interleukin-3 (IL-3), primary *FLT3*-mutant AML cells escaped FLT3 inhibitor-induced apoptosis [45]. The high CD123 expression in *FLT3*-ITD AML cases may result in resistance to FLT3 inhibitors, suggesting that combination of FLT3 inhibitors and CD123-targeted therapy may improve clinical responses in AML.

Like CD123, CD25 has also been investigated as a therapeutic target in hematologic malignancies. Denileukin diftitox, a fusion protein combining diphtheria toxin and interleukin-2 (IL-2), was effective in treating B- and T-cell lymphomas [46,47]. Treating T-cell lymphoma patients with daclizumab (an anti-CD25 monoclonal antibody) was reported to achieve good response [48,49]. CD25 is also an attractive therapeutic target for AML patients because it is not only expressed on leukemic cells but also on immunosuppressive Tregs. Tregs are increased in AML patients and the higher level of Tregs correlates with poorer patient outcomes [50,51,52]. Targeting CD25 may facilitate elimination of Tregs in addition to leukemic cells, restoring the immune permissive microenvironment. Treatment of primary AML patient cells with CD25 Mab, a human CD25 specific glycoengineered IgG1 antibody, led to the specific killing of two different cell types, CD25+ AML blasts and regulatory T cells [34].

The frequent high expression levels of CD25 and CD123 observed in *KMT2A*-PTD AML in this study suggests that agents targeting these antigens may be potential treatment strategies for these patients. In this study, 20 (43%) *KMT2A*-PTD AML cases showed both aberrant CD25 and increased CD123 expression, suggesting that dual targeting of CD25 and CD123 on these patients may be superior to single-agent approaches in these patients. One possible consequence of using CD123-targeting agents is the depletion of normal hematopoietic cells, since normal myeloblasts express CD123 (at low level). This side effect probably will not occur when using anti-CD25 therapy due to lack of CD25 on normal myeloblasts.

## 5. Conclusions

In summary, our study provides the first comprehensive immunophenotypic characterization and immunophenotype-genotype association study of *KMT2A*-PTD AML. Our results show that cases of AML with *KMT2A*-PTD show a different immunophenotype from *KMT2A*-rearranged AML. *KMT2A*-PTD AML cases most frequently have CD34+ CD117+ HLA-DR+ blasts and most often do not have monocytic differentiation. *KMT2A*-PTD AML cases are characterized by aberrant CD25 and increased CD123 expression, both of which are associated with *FLT3*-ITD. Our data suggests that CD25 and CD123 may serve as potential immunophenotypic biomarkers and therapeutic targets in patients with *KMT2A*-PTD AML. Importantly, we show that cases of AML with *KMT2A*-PTD differ greatly from cases of AML with *KMT2A* rearrangement. The sample size of this cohort is relatively small due to the rarity of *KMT2A*-PTD in AML. Larger studies are needed to confirm our findings.

## Figures and Tables

**Table 1:** Clinicopathologic and Molecular Characteristics of AML with *KMT2A*-PTD.

Characteristic (n = 47)	Value
Age, years (median, range)	67 (19–87)
Gender (Male/Female)	32/15 (2.1:1)
History of hematological malignancies	9 (19%)
Myelodysplastic syndrome	5 (11%)
Chronic myelomonocytic leukemia	3 (6%)
Myeloproliferative neoplasm	1 (2%)
Immunophenotype	
AML, granulocytic	31 (66%)
AML with granulocytic and/or monocytic differentiation	16 (34%)
Myelomonocytic	11 (23%)
Monoblastic	5 (11%)
Karyotype (n = 45)	
Normal or non-complex	42 (93%)
Complex	3 (7%)
Common recurrent mutations	
*FLT3*-ITD	22 (47%)
*DNMT3A*	20 (43%)
*RUNX1*	11 (23%)
*FLT3*-TKD	7 (15%)

## Data Availability

The datasets used and/or analyzed during the current study are available from the corresponding author on reasonable request.

## Supplementary Materials

The following supporting information can be downloaded at: https://www.mdpi.com/article/10.3390/cancers18020282/s1, Table S1. The product information of antibodies used in flow cytometric analysis. Table S2. The list of 81genes included in next generation sequencing panel. Table S3. The mutation profile of all KMT2A-PTD AML cases.
