# Supplementary material for: Aberrant CD25 and Increased CD123 Expression Are Common in Acute Myeloid Leukemia with KMT2A Partial Tandem Duplication and Are Associated with FLT3 Internal Tandem Duplication
Source: Cancers (Basel). 2026 Jan 16;18(2):282. doi: 10.3390/cancers18020282 (PMC12839231; doi:10.3390/cancers18020282)
Supplement: Supplementary file 1 [file cancers-18-00282-s001.zip › cancers-4070037-supplementary.pdf]

Supplemental Table 1. The product information of antibodies used in flow cytometric analysis

Tube	FITC	PE	PerCP-Cy5.5	PE-Cy7	APC	APC-R718	APC-H7	V450	V500	BV605	BV711	BV786
Tube 1-surface	CD7	CD117	CD34	CD33	CD13	CD38	CD45	CD133 BV421	CD5 BV510	CD4	CD54	CD19
Tube 2-surface	CD36	CD117	CD34	CD64	CD123	CD38	CD45	HLA-DR	CD15 BV510	CD56	CD25	CD14
Tube 3-perm	cTDT	cMPO	sCD34	sCD2	sCD19		sCD3	cCD3 BV421	sCD45	sCD117		

Markers	Fluorescent conjugate	Clone name	Company
CD2	PE-Cy7	L303.1	BD Biosciences
CD3 (cytoplasmic)	BV421	UCHT1	BD Biosciences
CD3 (surface)	APC-H7	SK7	BD Biosciences
CD4	BV605	SK3	BioLegend
CD5	BV510	UCHT2	BD Biosciences
CD7	FITC	4H9	BD Biosciences
CD13	APC	WM15	BD Biosciences
CD14	BV786	M5E2	BD Biosciences
CD15	BV510	W6D3	BD Biosciences
CD19	APC	SJ25C1	BD Biosciences
CD19	BV786	SJ25C1	BD Biosciences
CD25	BV711	2A3	BD Biosciences
CD33	PE-Cy7	P67.6	BD Biosciences
CD34	PerCP-Cy5.5	8G12	BD Biosciences
CD36	FITC	CLB-IVC7	BD Biosciences
CD38	R718	HB7	BD Biosciences
CD45	APC-H7	2D1	BD Biosciences
CD45	V500	HI30	BD Biosciences
CD54	BV711	HA58	BD Biosciences
CD56	BV605	B159	BD Biosciences
CD64	PE-Cy7	10.1	BioLegend
CD117	PE	104D2	BD Biosciences
CD117	BV605	104D2	BD Biosciences
CD123	APC	7G3	BD Biosciences
CD133	BV421	293C3	BD Biosciences
HLA-DR	V450	L243	BD Biosciences
MPO	PE	5B8	BD Biosciences
TDT	FITC	n/a	Supertechs

Supplemental Table 2. The list of 81genes included in next generation sequencing panel.

Gene	Exons (codons) tested
ANKRD26 (NM_014915)	1 (1-5)
ASXL1 (NM_015338)	12-13 (363-1290), 13 (1300-1443), 13 (1450-1542)
ASXL2 (NM_018263)	12-13 (381-1300), 13 (1312-1436)
BCOR (NM_017745)	2-4 (1-512), 4-15 (514-1644), 15 (1663-1722)
BCORL1 (NM_021946)	1-6 (1-1261), 6 (1292-1324), 6-8 (1327-1487), 9-12 (1492-1699), 12 (1706-1712)
BRAF (NM_004333)	11 (439-477), 15 (581-620)
BRINP3 (NM_199051)	2-8 (1-471), 8 (474-500), 8 (502-767)
CALR (NM_004343)	9 (352-418)
CBL (NM_005188)	7-9 (337-477)
CBLB (NM_170662)	7-10 (282-469)
CBLC (NM_012116)	7-9 (336-454), 10 (465-475)
CEBPA (NM_004364)	1 (1-56), 1 (59-90), 1 (274-359)
CREBBP (NM_004380)	1-8 (1-608), 9-10 (615-704), 12-16 (720-1083), 17-31 (1093-1943), 31 (1963-2042), 31 (2049-2443)
CRLF2 (NM_022148)	6 (230-234)
CSF3R (NM_156039)	14 (575-621), 17 (681-800), 17 (821-864)
CUX1 (NM_181552)	2-6 (11-173), 6-9 (175-241), 10-14 (248-408)
DDX41 (NM_016222)	1-10 (1-366), 11-17 (387-623)
DNMT3A (NM_022552)	8-22 (286-861), 23 (866-913)
EED (NM_003797)	1-2 (1-69), 2-8 (71-287), 9-12 (289-442)
ELANE (NM_001972)	1-2 (1-47), 2 (69-75), 4-5 (123-268)
ETNK1 (NM_018638)	3 (139-186)
ETV6 (NM_001987)	1-8 (1-453)
EZH2 (NM_004456)	2-5 (1-157), 5-6 (160-169), 7 (209-216), 8-13 (243-512), 14-15 (516-613), 15-19 (616-732), 20 (752)
FBXW7 (NM_033632)	9-12 (413-708)
FLT3 (NM_004119)	11-20 (437-847)
GATA1 (NM_002049)	2-3 (1-85)
GATA2 (NM_032638)	2-5 (1-377), 5 (379-381), 6 (383-481)
GFI1 (NM_005263)	2 (2-39)
GNAS (NM_000516)	8 (201-202), 11 (316-324)
HNRNPK (NM_002140)	3-7 (1-96), 8-17 (111-465)
HRAS (NM_005343)	2-3 (1-60), 3-4 (87-150)
IDH1 (NM_005896)	4 (64-132)
IDH2 (NM_002168)	4 (125-178)
IKZF1 (NM_006060)	2-8 (1-431), 8 (480-518)
IL2RG (NM_000206)	1-2 (1-45), 2-8 (51-340), 8 (359-370)
IL7R (NM_002185)	5-7 (180-292)
JAK1 (NM_002227)	3-22 (3-1023), 22-24 (1026-1123)
JAK2 (NM_004972)	10 (406-442), 12-14 (505-622), 16 (665-711), 18 (762-805)
JAK3 (NM_000215)	2-23 (1-1069)
KDM6A (NM_021140)	1-19 (1-971), 19-23 (976-1096), 23-29 (1098-1402)
KIT (NM_000222)	8-9 (411-514), 11 (550-592), 17 (788-828)
KMT2A (NM_005933)	2-4 (145-1075), 4-7 (1081-1335), 7-10 (1338-1441), 11-13 (1445-1561), 14-15 (1566-1665), 27 (2179-2195), 27 (2201-2362), 27 (2365-3217), 27 (3220-3327), 27 (3336-3582)
KRAS (NM_004985)	2-4 (1-150)
MAP2K1 (NM_002755)	2 (28-91), 3 (99-146)
MPL (NM_005373)	10 (490-522), 12 (552-636)
NF1 (NM_001042492)	2-5 (21-190), 6 (202-218), 8-9 (244-309), 9-13 (311-468), 13-14 (478-547), 15-17 (568-667), 18 (674-728), 18-21 (746-950), 23 (998-1038), 24-26 (1040-1146), 26-30 (1160-1370), 31-35 (1382-1550), 35 (1563-1575), 36-38 (1577-1869), 39-40 (1871-1947), 40-47 (1952-2323), 47-49 (2325-2439), 50-51 (2441-2491), 51 (2494-2539), 53-58 (2580-2840)
NOTCH1 (NM_017617)	26 (1529-1594), 26-28 (1601-1795), 34 (2069-2230), 34 (2234-2274), 34 (2290-2556)

NPM1 (NM_002520)	11 (283-295)
NRAS (NM_002524)	2-4 (1-150)
PAX5 (NM_016734)	1-10 (1-392)
PHF6 (NM_032458)	2-10 (1-366)
PIGA (NM_002641)	2 (1-5), 2-6 (15-485)
PML (NM_033238)	3 (201-256)
PRPF40B (NM_001031698)	2 (2-16), 2-19 (20-608), 19 (612-627), 20 (629-658), 20-26 (662-893)
PTEN (NM_000314)	7-8 (212-287)
PTPN11 (NM_002834)	3-4 (47-126), 7 (253-285), 12 (461-463), 12-13 (465-533)
RAD21 (NM_006265)	2-3 (1-81), 3 (83), 4-7 (92-249), 7-14 (260-632)
RARA (NM_000964)	6-7 (211-315)
RUNX1 (NM_001754)	2-9 (1-421), 9 (425-437)
SETBP1 (NM_015559)	4 (839-886)
SF1 (NM_004630)	1-2 (1-53), 3-12 (57-524), 13 (528-579), 13 (582-640)
SF3A1 (NM_005877)	1-9 (1-424), 9-12 (427-639), 13-16 (651-794)
SF3B1 (NM_012433)	13-16 (574-790)
SH2B3 (NM_005475)	2 (1-99), 2 (132-164), 2-8 (233-576)
SMC1A (NM_006306)	1-7 (1-415), 8-19 (419-983), 20-24 (992-1206), 25 (1210-1234)
SMC3 (NM_005445)	1 (1-5), 2-6 (20-110), 7-16 (118-505), 16-17 (508-580), 17-20 (592-727), 20-27 (729-1151), 28-29 (1159-1218)
SRSF2 (NM_003016)	1 (1-37), 1 (44-121)
STAG1 (NM_005862)	2 (1-5), 3 (10-27), 4-12 (45-392), 13-20 (402-703), 21-22 (717-737), 22-29 (739-1049), 29-34 (1051-1259)
STAG2 (NM_006603)	2-16 (1-518), 16-33 (541-1232)
STAT3 (NM_139276)	17 (489-503), 17-22 (521-715)
STAT5A (NM_003152)	3-7 (1-215), 7-20 (224-795)
STAT5B (NM_012448)	16 (636-673)
SUZ12 (NM_015355)	1 (46-84), 2-5 (92-169), 6-16 (180-740)
TERC (NR_001566)	coordinate 169,482,148 - 169,483,176
TERT (NM_198253)	1 (1-24), 2 (80-165), 2 (257-300), 2 (311-342), 2-4 (349-630), 4-5 (633-677), 6-16 (711-1133)
TET2 (NM_001127208)	3 (1-77), 3 (91-826), 3 (829-853), 3 (867-1027), 3-10 (1029-1455), 10-11 (1465-2003)
TP53 (NM_000546)	2 (1-25), 4-11 (79-394)
U2AF1 (NM_006758)	2 (15-44), 6 (117-161)
U2AF2 (NM_007279)	1 (1-17), 3-5 (63-138), 5 (140-161), 6-12 (163-437)
WT1 (NM_024426)	1 (7-49), 1 (127-171), 1-10 (192-523)
ZRSR2 (NM_005089)	1-4 (1-90), 6-8 (134-213), 8 (215-257), 10-11 (277-435), 11 (465-483)

Supplemental Table 3. The mutation profile of all *KMT2A*-PTD AML cases.

Case ID	Mutations
1	*NF1, U2AF1, BCOR1, FLT3-ITD*
2	*ETV6, IDH2*
3	*DNMT3A, RUNX1, SRSF2, FLT3-ITD*
4	*NRAS, FLT3-ITD*
5	*DNMT3A, FLT3-ITD*
6	*FLT3-TKD, PHF6, RUNX1, WT1*
7	*DNMT3A, IDH1, STAG2*
8	*ASXL1, NRAS, PTPN11*
9	*IDH2, STAG2*
10	*DNMT3A, IDH2, FLT3-ITD*
11	*DNMT3A, RUNX1, SF3B1, FLT3-ITD*
12	*RUNX1, IDH2, FLT3-ITD*
13	*DNMT3A, IDH2*
14	*ASXL1, RUNX1*
15	*ASXL1, SRSF2, STAG2, TET2*
16	*DNMT3A, NRAS, TET2, WT1*
17	*U2AF1*
18	*IDH2, TET2, TP53, WT1, JAK2, FLT3-ITD*
19	*TP53, U2AF1, ETV6, NRAS, FLT3-ITD*
20	*DNMT3A, BCOR, KRAS, U2AF1, NRAS*
21	*BCOR, NF1, TET2, U2AF1*
22	*BCOR, NRAS, RUNX1, TP53*
23	*DNMT3A, U2AF1, FLT3-TKD*
24	*TET2, U2AF1*
25	*DNMT3A, SF3B1, WT1*
26	*ASXL1, IDH2, RUNX1, SRSF2, STAG2, TET2*
27	*DNMT3A, SF3B1, FLT3-ITD*
28	*CBL, FLT3-TKD, PHF6, RUNX1, STAG2, FLT3-ITD*
29	*DNMT3A, SRSF2, TET2, ASXL1, JAK2*
30	*DNMT3A, WT1, FLT3-ITD*
31	*STAG2, TET2, WT1, FLT3-ITD*
32	*DNMT3A, TET2, U2AF1, CBL, FLT3-ITD*
33	*DNMT3A, PHF6, WT1, FLT3-TKD*
34	*ETV6, IDH2, WT1*
35	*DNMT3A, IDH2, TP53, FLT3-ITD*

36	*DNMT3A, BCOR, WT1, FLT3-ITD*
37	*DNMT3A, BCOR, IDH1, RUNX1*
38	*TET2, U2AF1*
39	*ASXL2, FLT3-TKD (p.N676K), IDH1, NF1, RUNX1, SRSF2, STAG2*
40	*TET2, FLT3-ITD*
41	*FLT3-TKD, WT1, TET2*
42	*NRAS, U2AF1, TET2*
43	*PHF6, STAG2, WT1*
44	*DNMT3A*
45	*BCOR, FLT3-TKD, RUNX1, SRSF2*
46	*ASXL1, TET2*
47	*WT1*
